# Game of Mirrors: Health Profiles in Patient and Physician Perceptions

**DOI:** 10.3390/ijerph19031201

**Published:** 2022-01-21

**Authors:** Daniele Fineschi, Sofia Acciai, Margherita Napolitani, Giovanni Scarafuggi, Gabriele Messina, Giovanni Guarducci, Nicola Nante

**Affiliations:** 1 Local Health Unit Tuscany South-East, 53100 Siena, Italy; doc.fineschi@gmail.com (D.F.); acciaisofia@gmail.com (S.A.); 2 Post Graduate School of Public Health, University of Siena, 53100 Siena, Italy; napolitani2@student.unisi.it (M.N.); gabriele.messina@unisi.it (G.M.); giovanni.guarducc@student.unisi.it (G.G.); 3 Department of Medicine, Surgery and Neuroscience, University of Siena, 53100 Siena, Italy; g.scarafuggi@gmail.com; 4 Department of Molecular and Developmental Medicine, University of Siena, 53100 Siena, Italy

**Keywords:** empathy, euroqol, health-related quality of life, general practitioner, patient different perception

## Abstract

The relationship between general practitioners and patients is privileged. The aim of this study was to assess the concordance between the health profile perceived by the patient and the one described by the doctor. We conducted a cross-sectional study between 2019–2020. Patients completed the 5d-5L (EQ-p) and clinicians completed it “from the patient’s perspective” (EQ-d), also consulting the clinical diary. Statistical analysis was performed using Stata 14 (Cohen’s kappa; Fisher’s exact test). The sample consisted of 423 patients. The mean age was 56.7 ± 19.2. There were significant differences by gender in usual activities, pain, and anxiety/depression (74.6% of men had no limitation in usual activities versus 64.5% of women (*p* < 0.01), 53.9% of men had no pain versus 38.5% of women (*p* < 0.01), and 60.3% of men had no anxiety/depression versus 38.5% of women (*p* < 0.01)). Physicians did not detect these differences. The concordance between EQ-p and EQ-d was substantial for mobility (k = 0.62; *p* < 0.01), moderate for self-care (k = 0.48; *p* < 0.01) and usual activities (k = 0.50; *p* < 0.01). Concordance was fair for pain/discomfort (k = 0.32; *p* < 0.01), anxiety/depression (k = 0.38; *p* < 0.01), and EQ Index (k = 0.21; *p* < 0.01). There was greater agreement for “objective “dimensions (mobility, self-care, and usual activities). A good doctor, to be considered as such, must try to put himself in the “patient’s pajamas” to feel his feelings and be on the same wavelength.

## 1. Introduction

Health is a multidimensional parameter not only related to bio-physiopathology. Nowadays psycho-socio-economic factors are considered as an integral part of citizen health in the context of advanced health policies [1,2]. This change in attitude has fostered the development of methods of measuring the phenomenon that value the individual’s perception of his condition, which is not limited to a mere analysis of clinical features [3]. These tools are increasing [4,5,6,7,8] and adapting to various purposes [9], from the generic ones to the most specific and refined. The concept of health, therefore, has been partly merged, and partly extended, into the much broader concept of quality of life [2,10].

Alongside this paradigm change, there has been a shift in chronicity, with the lengthening of life expectancy and the increase in pathologies for which there is no longer any talk of recovery, but of management (often long and costly). Since health economic policy decisions can no longer be guided only by the survival target, tools are needed to measure the quality of life gained, such as the Quality Adjusted Life Year (QALY) [11,12]. In fact, it is a weighting index of expected life years, used for cost-utility analyses of healthcare interventions. To calculate the QALY, perceived health measurement tools are used, among which one of the best known is the EuroQol-5D Questionnaire (EQ 5D-5L) [12,13]; this is a generic measure of Health-Related Quality of Live (HRQoL), or health-related quality of life [1,4,7,14,15,16].

However, it is fair to question whether this tool can find a place in everyday clinical practice [10]. As physicians, we are educated to take care of our patients’ health by treating their alterations (problem-oriented approach), which we must first look for and diagnose, but it is increasingly evident that a method focused instead on the patient as a whole provides better results [17]. It is a common habit for physicians not to give too much weight to the “self-diagnosis” proposed by the patient, and even to distrust it in order not to run into mistakes; however, by doing so, they are hardly able to understand the needs of a patient when they go beyond the purely clinical [18,19]. The doctor-patient conversation, after all, is unbalanced both culturally and in terms of decision-making [20]. However, in a world where health is no longer related only to disease, this therapeutic diagnostic process must be re-weighted [18].

From this point of view, various attempts have been made to measure how much physicians are really able to understand the broader health of those in front of them; actual scales to measure empathy [21,22]. The general practitioner (or family physician) perhaps lends itself to this type of reasoning more than others [23]: inserted in the territory, in a prolonged and lasting contact with patients and all their problems (not only health), is generally able to understand a patient’s full story. By vocation, a general practitioner does not focus on a single apparatus or a single pathology. That is why we conducted an identification exercise by asking the physician and the patient to describe, independently and using the same assessment test (EQ 5D-5L), the latter’s perception of health. The patient was asked to fill out the usual form, while the doctor was asked to try to interpret the perception of the person in front of him (“wearing his shoes”, to borrow an Anglo-Saxon expression), having available not only the “reported” clinical information but also the “objective” information, both recent and previous, concerning him.

It is essential to investigate the ability of physicians to empathize with their patients because it is known that the empathic physician receives greater gratification in his work, decreasing the risk of burnout, while the patient, feeling understood, will have more confidence in the physician and there will be an increase in compliance [24].

The aim of this work was to evaluate the concordance of the two survey techniques conducted with the same instrument (EQ 5D-5L): the health perceived by the patient and the health profile described by the physician for that same patient.

## 2. Materials and Methods

**Figure 1:**
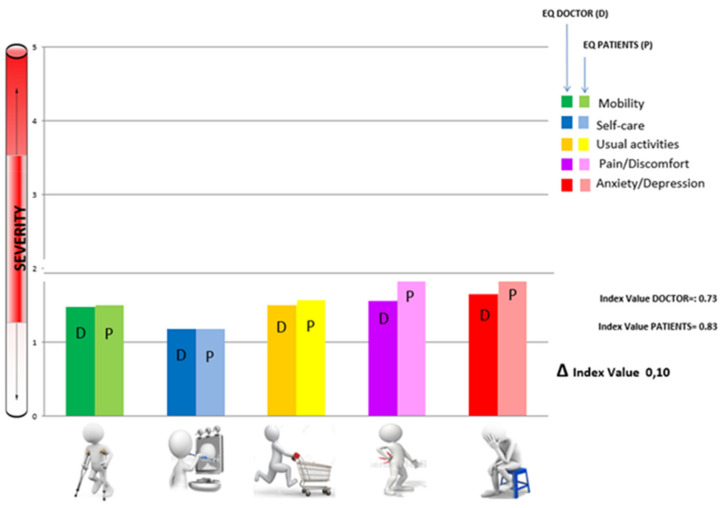
Average values EQ-5D-5L expressed, on the same clinical casuistry, by physicians and patients.

In the Period from July 2019 to December 2020, we conducted a cross-sectional study using the EQ 5D-5L questionnaire (Italian version, Annex A) and the EQ-5D-5L index (EQ Index). The questionnaire investigates five dimensions: mobility, self-care, usual activities, pain/discomfort, and anxiety/depression. For each dimension there are 5 levels: no problems, mild problems, moderate problems, severe problems, and extreme problems [14,15]. The EQ Index is a HRQoL sp (0-core obtained by applying a weighted value) to each dimension score. Indexes scores range from −1 (health status worse than death) to 1 (perfect health) [16]. During our research, the EQ 5D-5L questionnaires was submitted to 423 patients in a general medicine and continuity of care setting. At the same time, the same physicians or medical student completed a similar form for each patient, based on the history, the computerized clinical diary of their management software, and a physical examination. Our sample consisted of patients who casually went to the general practitioner’s office. They were not end of life patients and were not patients requiring hospitalization for acute diseases. The results of the two 5D-5L EQs (renamed EQ-Patients and EQ-Physicians) thus compiled for each patient were entered into a database using the EXCEL Software and then were compared by means of an agreement test (Cohen’s Kappa) and Fisher’s exact test using the STATA Software SE/14.0 (StataCorp LLC, Station, TX, USA). Cohen’s kappa (κ) is a measure of inter-rater agreement for categorical scales when there are two raters. To interpret the results of Cohen’s kappa we referred to the following intervals: from 0.01 to 0.20 slight agreement, from 0.21 to 0.40 fair agreement, from 0.41 to 0.60 moderate agreement, from 0.61 to 0.80 substantial agreement and from 0.81 to 1.00 almost perfect or perfect agreement [25]. Our project has been registered and authorized by the EuroQol group (ID 30839 e ID 30840). The results are reported graphically on a model that we have elaborated ad hoc to make the reading of the health profiles obtainable with EQ-5D-5L more immediate (Figure 1).

## 3. Results

Our sample consisted of 423 patients, 55% of whom were women. The mean age was 56.7 ± 19.2 years; the youngest was 18 years old, and the oldest was 95 years old.

Table 1 shows the five dimension of patients’ EQ-5D-5L questionnaire divided by gender. By analyzing the answers from the EQ 5D-5L questionnaire, we found statistically significant differences between the men and women in our sample in both their perception of usual activities, physical pain, and psychological state. Regarding the usual activities, 74.6% of men reported no limitation of usual activities versus 64.5% of women. In addition, 7.3% of women and 0.5% of men reported severe or extreme limitation (Fisher’s exact < 0.01). Moreover, 53.9% of men and 38.5% of women reported feeling no pain and 6.8% of women reported experiencing severe or extreme pain, which was reported by only 1.1% of men (Fisher’s exact < 0.01). According to the anxiety/depression dimension, men seemed to have less distress than women: 60.3% of men, in fact, reported being neither anxious nor depressed, compared with 38.5% of women. In contrast, 26.1% of women felt from moderately to extremely anxious/depressed, compared with 11.1% of men. (Fisher’s exact < 0.01). No statistically significant differences were found for the other dimensions (mobility and self-care).

Table 2 shows the physician’s EQ-5D-5L. The general practitioners, in their exercise of empathy, failed to highlight the above-mentioned gender differences. In fact, in the opinion of the physicians, it was mostly men who felt no pain (65.1% vs. 56.8%), but this difference was not statistically significant (Fisher’s exact = 0.16). Moreover, the percentage of women and men who seemed to feel extremely anxious or depressed was not statistically significant (8.6% of women vs. 6.4% of men; Fisher’s exact = 0.06).

Table 3 shows the level of agreement (Cohen’s kappa) between EQ-Physician (EQ-d) and EQ-Patient (EQ-p), whose average values are expressed in Figure 1. The level of agreement between EQ-p and EQ-d was substantial for mobility (k = 0.62; *p* < 0.01), moderate for usual activities (k = 0.50; *p* < 0.01), and self-care (k = 0.48; *p* < 0.01). Finally, we found fair agreement for the dimension of physical pain (k = 0.32; *p* < 0.01), anxiety/depression (k = 0.38; *p* < 0.01), and EQ Index (k = 0.21 *p* < 0.01).

Table 4 shows the level of agreement (Cohen’s kappa) between EQ-Physician (EQ-d) and EQ-Patient (EQ-p) divided by gender. The level of agreement between EQ-p and EQ-d was higher for females for the dimension of mobility (substantial for females and moderate for males) and self-care (moderate for female and fair for males). For the other three dimensions there was the same level of agreement: moderate for usual activities and fair for pain/discomfort and anxiety/depression.

## 4. Discussion

We found no other studies in the literature with results directly comparable to the present study. While there are indeed growing interests in the study of physician empathy toward the patient [19,21,26,27,28], these studies are generally conducted with specially designed psychometric instruments: the Interpersonal Relativity Index (IRI), the Jefferson Scale of Physician Empathy (JSPE), the Jefferson Scale of Patient Perception of Physician Empathy (JSPPPE), the Consultation and Relational Empathy (CARE) scale, and others.

In 2007, Neumann et al. [26] studied the correlation between empathy as measured by the CARE scale and HRQoL outcomes (measured by self-administered tests other than EQ 5D-5L) over time in a cohort of cancer patients, showing a correlation between better physician empathy and better HRQoL outcomes. However, an outcome assessment was not an aim of our study.

Very popular is the approach with the IRI scale [22,27,28,29], one of the most widely used instruments in this field: created to assess the general population and not specifically healthcare personnel, it presents 28 items with 5 levels each, divided into subcategories (fantasy, perspective taking, empathic concern, personal distress). However, it is a generic and nonspecific tool to assess an individual’s empathy, whereas in our case we used an HRQoL assessment tool. Lucas Molina et al. [29] also suggest that women usually obtain a better IRI score than men, thus indicating greater empathy.

The Jefferson scale is another fairly widespread instrument in the study of empathy [19,21,22,27,28], specifically in the medical field: the first version, the JSPE, was developed by a group of 36 family physicians, and is a 20-item questionnaire of 7 levels each, with 3 subscales (sharing perspectives, compassionate care and being in the patient’s shoes), which the physician self-administers.

The same working group later also proposed the patient-filling version (the target is always physician empathy), or JSPPPE, consisting of 5 items of 7 levels each, to verify the reliability of the first scale.

The results obtained by Bernardo et al. [19,27] are also interesting: assuming that empathy is now a tool to improve clinical outcomes for the patient but also the physician’s work [30,31], his team investigated whether the tests with which the physician measures his empathy are associated with the results of those in which the patient judges the professional. They showed that self-administered empathy measurement scales (IRI and JSPE) did not correlate significantly with outcome improvement. In contrast, scales by which the patient measures empathy received during the visit (CARE and JSPPPE) correlated directly with better outcomes, clinical and otherwise. Thus, the medical staff studied did not seem to have an awareness of their own level of empathy. In our study, therefore, the use of the EQ-5D-5L questionnaire proved useful because it allowed us to assess and make the physician aware of his or her level of empathy.

Our experiment, like “a game”, aimed to evaluate and measure whether the participating doctors were able to identify themselves with the patients, also knowing in detail their clinical situation. This has occurred sufficiently in the dimensions of health that are more easily objectified, but not in the dimensions that require a greater empathy and understanding of the feelings: in fact, there was low agreement between the answers given by the participating doctors and those of the patients, especially in the dimensions more related to the psychological sphere pain/discomfort (k = 0. 14; *p* < 0.01) and anxiety/depression (k = 0.19; *p* < 0.01), as well as in the EQ Index (k = 0.11 *p* < 0.01).

Our analysis did not consider any collection of objective clinical data, since this was not among our objectives, so it is difficult to account for the variations in HRQoL between the various categories of patients. In our opinion, the significant difference found in the scores obtained by women and men in the dimensions pain/discomfort and anxiety/depression (mainly because the participating physicians failed to detect it) deserves further investigation. We can try to formulate hypotheses: the values reported in the tests by men, for example, could undergo a cultural bias of “social expectancy” (social desirability bias, [29]), where the male fills out the test following what he socially considers acceptable for his category, and therefore not reporting pain and inner discomfort. In this scenario, women would instead be able to present a more realistic self-analysis. Bias would be diminished in the physician’s version, who knows the patient’s clinic. Conversely, it may be the women who exaggerate. Finally, it is possible that participating physicians may have unintentionally tended to “flatten” the results, due to lack of empathy, limited time, poor listening skills, or high job stress. To test this, it would be necessary to repeat the experiment, increasing both the number of physicians and patients enrolled.

It is fair to point out that this study was affected by the limited number of patients involved, and, even more so, by the limited number of surveyors, which were only three. Among the various problems faced, perhaps the greatest obstacle encountered in the organizational phase was complying with the European Law for the Protection of Privacy and Sensitive Data, which slowed down the conduction of the study.

It is also important to consider that surveyors, being young people participating in the new structure of Territorial Functional Aggregations (AFT) or being medical students in extended internships with a general practitioner, did not have long-standing retrospective knowledge of the patients.

## 5. Conclusions

In our study, the EQ-5D-5L proved to be a good tool for assessing physician-patient empathy. It allowed participating physicians to empathize well with patients for the “more objective” dimensions (mobility, self-care, and usual activities). However, physicians showed good observational skills but less empathy in the dimensions most influenced by the psychological sphere (pain/discomfort and anxiety/depression). A good doctor, to be considered as such, must try to put himself in the “patient’s shoes” to feel his feelings and be on the same wavelength. Empathy can improve the doctor-patient relationship, benefiting both the doctor and the patient.

## Figures and Tables

**Table 1:** Patients’ five dimensions of EQ 5D 5L divided for gender.

	Mobility									
	None		Slight		Moderate		Severe		Extreme	
	N°	%	N°	%	N°	%	N°	%	N°	%
FEMALES	164	70.1	42	17.9	14	6	10	4.3	4	1.7
MALES	139	73.5	31	16.5	14	7.4	5	2.6	0	0
Fisher’s exact = 0.4										
	**Self-Care**									
	**None**		**Slight**		**Moderate**		**Severe**		**Extreme**	
	**N°**	**%**	**N°**	**%**	**N°**	**%**	**N°**	**%**	**N°**	**%**
FEMALES	188	80.4	23	9.8	13	5.6	5	2.1	5	2.1
MALES	162	85.7	19	10.1	7	3.7	1	0.5	0	0
Fisher’s exact = 0.1										
	**Usual Activities**									
	**None**		**Slight**		**Moderate**		**Severe**		**Extreme**	
	**N°**	**%**	**N°**	**%**	**N°**	**%**	**N°**	**%**	**N°**	**%**
FEMALES	151	64.5	44	18.8	22	9.4	10	4.3	7	3
MALES	141	74.6	34	18	13	6.9	0	0	1	0.5
Fisher’s exact < 0.01										
	**Pain/Discomfort**									
	**None**		**Slight**		**Moderate**		**Severe**		**Extreme**	
	**N°**	**%**	**N°**	**%**	**N°**	**%**	**N°**	**%**	**N°**	**%**
FEMALES	89	38	73	31.3	56	23.9	11	4.7	5	2.1
MALES	102	53.9	55	29.1	30	15.9	2	1.1	0	0
Fisher’s exact < 0.01										
	**Anxiety/Depression**									
	**None**		**Slight**		**Moderate**		**Severe**		**Extreme**	
	**N°**	**%**	**N°**	**%**	**N°**	**%**	**N°**	**%**	**N°**	**%**
FEMALES	90	38.5	83	35.5	48	20.5	9	3.8	4	1.7
MALES	114	60.3	54	28.6	19	10.1	0	0	2	1
Fisher’s exact < 0.01										

**Table 2:** EQ-5D 5L-Physician point of view-Pain/Discomfort and Anxiety/Depression.

	P-Pain/Discomfort									
	None		Slight		Moderate		Severe		Extreme	
	N°	%	N°	%	N°	%	N°	%	N°	%
FEMALES	133	56.8	56	23.9	39	16.8	5	2.1	1	0.4
MALES	123	65.1	45	23.8	18	9.5	3	1.6	0	0
Fisher’s exact = 0.16										
	**P-Anxiety/Depression**									
	**None**		**Slight**		**Moderate**		**Severe**		**Extreme**	
	**N°**	**%**	**N°**	**%**	**N°**	**%**	**N°**	**%**	**N°**	**%**
FEMALES	116	49.6	61	26	37	15.8	17	7.3	3	1.3
MALES	120	63.5	39	20.6	18	9.5	10	5.3	2	1.1
Fisher’s exact = 0.06										

**Table 3:** Level of agreement between EQ-Physician and EQ-Patient.

	Agreement	Expected Agreement	Kappa	Standard Error	Z	Prob > Z
Mobility	83.7%	56.7%	0.62	0.03	18.19	<0.01
Self-Care	83.7%	68.9%	0.48	0.04	13.38	<0.01
Usual activities	76.6%	53.5%	0.50	0.03	14.73	<0.01
Pain/discomfort	57.2%	37.4%	0.32	0.03	9.74	<0.01
Anxiety/depression	60.52%	36.8%	0.38	0.03	11.70	<0.01
EQ INDEX	30.73%	12.6%	0.21	0.01	14.61	<0.01

**Table 4:** Level of agreement between EQ-Physician and EQ-Patient divided by gender.

	MALES						FEMALES				
	Agreement	Expected Agreement	Kappa	Standard Error	Z	Prob > Z	Agreement	Expected Agreement	Kappa	Standard Error	Prob > Z
Mobility	82.5%	58.6%	0.58	0.05	11.0	<0.01	84.6%	55.3%	0.66	14.5	<0.01
Self-Care	83.1%	72.5%	0.38	0.06	6.8	<0.01	84.2%	66.1%	0.53	11.7	<0.01
Usual activities	76.7%	59.1%	0.43	0.05	8.0	<0.01	76.5%	49.2%	0.54	12.4	<0.01
Pain/discomfort	64.6%	43.6%	0.37	0.05	7.1	<0.01	51.3%	33.2%	0.27	6.5	<0.01
Anxiety/depression	65.6%	45.2%	0.37	0.05	7.3	<0.01	56.4%	31.9%	0.36	8.8	<0.01
EQ INDEX	36.5%	16.7%	0.24	0.02	10.1	<0.01	26.1%	9.7%	0.18	10.5	<0.01

## Data Availability

The data are available from the authors on the departmental server of the University of Siena, 53100-Siena, Italy.

## References

[1] Mulhern B., Norman R., De Abreu Lourenco R., Malley J., Street D., Viney R. (2019). Investigating the relative value of health and social care related quality of life using a discrete choice experiment. Soc. Sci. Med..

[2] Moons P. (2004). Why Call it Health-Related Quality of Life When You Mean Perceived Health Status?. Eur. J. Cardiovasc. Nurs..

[3] Stolk E.A., Oppe M., Scalone L., Krabbe P.F.M. (2010). Discrete Choice Modeling for the Quantification of Health States: The Case of the EQ-5D. Value Health.

[4] Quercioli C., Messina G., Barbini E., Carriero G., Fanì M., Nante N. (2009). Importance of sociodemographic and morbidity aspects in measuring health-related quality of life: Performances of three tools: Comparison of three questionnaire scores. Eur. J. Health Econ..

[5] Nante N., Gialluca L., De Corso M., Troiano G., Verzuri A., Messina G. (2016). Quality of life in refugees and asylum seekers in Italy: A pilot study. Ann. Dell Istituto Super. Di Sanita.

[6] Kozloff N., Pinto A.D., Stergiopoulos V., Hwang S.W., O’Campo P., Bayoumi A.M. (2019). Convergent validity of the EQ-5D-3L in a randomized-controlled trial of the Housing First model. BMC Health Serv. Res..

[7] Longworth L., Rowen D. (2013). Mapping to Obtain EQ-5D Utility Values for Use in NICE Health Technology Assessments. Value Health.

[8] Strada L., Vanderplasschen W., Buchholz A., Schulte B., Muller A.E., Verthein U., Reimer J. (2017). Measuring quality of life in opioid-dependent people: A systematic review of assessment instruments. Qual. Life Res..

[9] Scoggins J.F., Patrick D.L. (2009). The use of patient-reported outcomes instruments in registered clinical trials: Evidence from ClinicalTrials.gov. Contemp. Clin. Trials.

[10] Moons P., Budts W., De Geest S. (2006). Critique on the conceptualisation of quality of life: A review and evaluation of different conceptual approaches. Int. J. Nurs. Stud..

[11] Messina G., Quercioli C., Troiano G. (2016). Italian medical students quality of life: Years 2005–2015. Ann. Hyg. Prev. Community Med..

[12] Dakin H., Abel L., Burns R., Yang Y. (2018). Review and critical appraisal of studies mapping from quality of life or clinical measures to EQ-5D: An online database and application of the MAPS statement. Health Qual. Life Outcomes.

[13] Buchholz I., Janssen M.F., Kohlmann T., Feng Y.-S. (2018). A Systematic Review of Studies Comparing the Measurement Properties of the Three-Level and Five-Level Versions of the EQ-5D. PharmacoEconomics.

[14] Devlin N.J., Brooks R. (2017). EQ-5D and the EuroQol Group: Past, Present and Future. Appl. Health Econ. Health Policy.

[15] Luo N., Liu G., Li M., Guan H., Jin X., Rand K. (2017). Estimating an EQ-5D-5L Value Set for China. Value Health.

[16] Silva A., Cancela J., Mollinedo I., Camões M., Bezerra P. (2021). The Relationship between Health Perception and Health Predictors among the Elderly across European Countries. Int. J. Environ. Res. Public Health.

[17] Dwamena F., Holmes-Rovner M., Gaulden C.M., Jorgenson S., Sadigh G., Sikorskii A., Lewin S., Smith R.C., Coffey J., Olomu A. (2012). Interventions for providers to promote a patient-centred approach in clinical consultations. Cochrane Database Syst. Rev..

[18] Purkaple B.A., Mold J.W., Chen S. (2016). Encouraging Patient-Centered Care by Including Quality-of-Life Questions on Pre-Encounter Forms. Ann. Fam. Med..

[19] Bernardo M.O., Cecilio-Fernandes D., de Abreu Lima A.R., Silva J.F., Ceccato H.D., Costa M.J., de Carvalho-Filho M.A. (2019). Investigating the relation between self-assessment and patients’ assessments of physicians-in-training empathy: A multicentric, observational, cross-sectional study in three teaching hospitals in Brazil. BMJ Open.

[20] Couture É.M., Chouinard M.-C., Fortin M., Hudon C. (2017). The relationship between health literacy and quality of life among frequent users of health care services: A cross-sectional study. Health Qual. Life Outcomes.

[21] Di Lillo M., Cicchetti A., Scalzo A.L., Taroni F., Hojat M. (2009). The Jefferson Scale of Physician Empathy: Preliminary Psychometrics and Group Comparisons in Italian Physician. Acad. Med..

[22] Hojat M., Gonnella J.S. (2017). What Matters More About the Interpersonal Reactivity Index and the Jefferson Scale of Empathy? Their Underlying Constructs or Their Relationships with Pertinent Measures of Clinical Competence and Patient Outcomes?. Acad. Med..

[23] Mercer S.W., Reynolds W.J. (2002). Empathy and quality of care. Br. J. Gen. Pract..

[24] Lee M., Noh Y., Youm C., Kim S., Park H., Noh B., Kim B., Choi H., Yoon H. (2021). Estimating Health-Related Quality of Life Based on Demographic Characteristics, Questionnaires, Gait Ability, and Physical Fitness in Korean Elderly Adults. Int. J. Environ. Res. Public Health.

[25] Landis J.R., Koch G.G. (1977). The measurement of observer agreement for categorical data. Biometrics.

[26] Neumann M., Wirtz M.A., Bollschweiler E., Mercer S., Warm M., Wolf J., Pfaff H. (2007). Determinants and patient-reported long-term outcomes of physician empathy in oncology: A structural equation modelling approach. Patient Educ. Couns..

[27] Bernardo M.O., Cecílio-Fernandes D., Costa P., Quince T.A., Costa M.J., Carvalho-Filho M.A. (2018). Physicians’ self-assessed empathy levels do not correlate with patients’ assessments. PLoS ONE.

[28] Cánovas L., Carrascosa A.-J., García M., Fernández M., Calvo A., Monsalve V., Soriano J.-F., Empathy Study Group (2018). Impact of Empathy in the Patient-Doctor Relationship on Chronic Pain Relief and Quality of Life: A Prospective Study in Spanish Pain Clinics. Pain Med..

[29] Lucas-Molina B., Pérez-Albéniz A., Ortuño-Sierra J. (2017). Dimensional structure and measurement invariance of the Interpersonal Reactivity Index (IRI) across gender. Psicothema.

[30] Silva R.G., Figueiredo-Braga M. (2018). The roles of empathy, attachment style, and burnout in pharmacy students’ academic satisfaction. Am. J. Pharm. Educ..

[31] Kim K. (2018). To Feel or Not to Feel: Empathy and Physician Burnout. Acad. Psychiatry.

